# {4′-[4-(4,4′-Bipyridin-1-ylmeth­yl)phen­yl]-2,2′:6′,2′′-terpyridine}chloridoplatinum(II) bis­(perchlorate) acetonitrile disolvate sesquihydrate

**DOI:** 10.1107/S1600536810017782

**Published:** 2010-05-22

**Authors:** Guiju Zhang, Quan Li, Wenfu Fu, Daqi Wang

**Affiliations:** a Technical Institute of Physics and Chemistry, Chinese Academy of Sciences, Beijing 100190, People’s Republic of China; b College of Chemistry and Chemical Engineering, Liaocheng University, Shandong 252059, People’s Republic of China

## Abstract

The asymmetric unit of the title compound, [PtCl(C_32_H_24_N_5_)](ClO_4_)_2_·2CH_3_CN·1.5H_2_O, comprises two unique Pt^II^ complex cations, four perchlorate anions, four acetonitrile solvent mol­ecules and three water mol­ecules. The Pt atom is four-coordinated by a tridentate chelating 2,2′:6′,2′′-terpyridine ligand and a chloride ion in a square-planar geometry with modest distortion imposed by the constraint of the terpyridyl ligand. The r.m.s. deviations from the plane comprising the four ligand donor atoms and the Pt atom are 0.0381 and 0.0472 Å in the two complex cations.

## Related literature

For the synthesis of the terpyridyl ligand, see: Kronke (1976 ▶); Collin *et al.* (1991 ▶). For the synthesis of Pt^II^–terpyridyl complexes, see: Jarosz *et al.* (2008 ▶). For the structures of similar complexes, see: Chakraborty *et al.* (2005 ▶); Sakai *et al.* (2003 ▶).
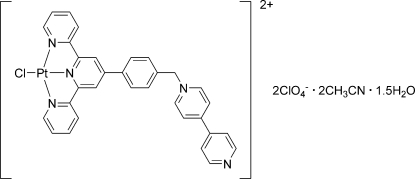


## Experimental

### 

#### Crystal data

[PtCl(C_32_H_24_N_5_)](ClO_4_)_2_·2C_2_H_3_N·1.5H_2_O
*M* *_r_* = 1017.14
Monoclinic, 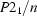

*a* = 13.3990 (15) Å
*b* = 14.5018 (15) Å
*c* = 41.279 (4) Å
β = 90.390 (1)°
*V* = 8020.8 (14) Å^3^
*Z* = 8
Mo *K*α radiation
μ = 3.76 mm^−1^
*T* = 298 K
0.20 × 0.19 × 0.18 mm

#### Data collection

Bruker SMART diffractometer
Absorption correction: multi-scan (*SADABS*; Sheldrick, 1996 ▶) *T*_min_ = 0.520, *T*_max_ = 0.551
41405 measured reflections
14131 independent reflections
5677 reflections with *I* > 2σ(*I*)
*R*_int_ = 0.143

#### Refinement

*R*[*F* ^2^ > 2σ(*F* ^2^)] = 0.114
*wR*(*F* ^2^) = 0.339
*S* = 1.03
14131 reflections
1022 parameters
H-atom parameters constrained
Δρ_max_ = 3.13 e Å^−3^
Δρ_min_ = −1.62 e Å^−3^

### 

Data collection: *SMART* (Siemens, 1996 ▶); cell refinement: *SAINT* (Siemens, 1996 ▶); data reduction: *SAINT*; program(s) used to solve structure: *SHELXS97* (Sheldrick, 2008 ▶); program(s) used to refine structure: *SHELXL97* (Sheldrick, 2008 ▶); molecular graphics: *ORTEP-3* (Farrugia, 1997 ▶) and *DIAMOND* (Brandenburg, 1998 ▶); software used to prepare material for publication: *SHELXTL* (Sheldrick, 2008 ▶).

## Supplementary Material

Crystal structure: contains datablocks I, global. DOI: 10.1107/S1600536810017782/sj2800sup1.cif

Structure factors: contains datablocks I. DOI: 10.1107/S1600536810017782/sj2800Isup2.hkl

Additional supplementary materials:  crystallographic information; 3D view; checkCIF report

## Figures and Tables

**Fig. 1.**
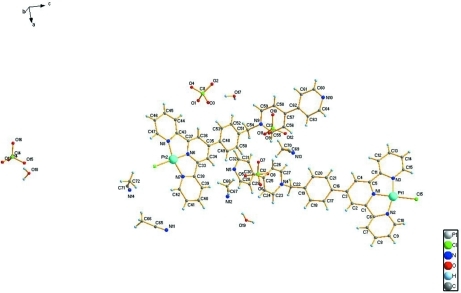
The asymmetric unit of the title compound with ellipsoids drawn at the 30% probability level.

## supplementary crystallographic information

### Comment

The molecular structure of the title compound is shown in Fig. 1. The complex consists of a distorted square-planar geometry around the Pt^II^ center with three coordination sites occupied by the terpyridyl ligand and the fourth taken up by the chloride ion. The rms deviation from a plane comprising the four ligand donor atoms and the Pt atom is 0.0381. The Pt-N distance of the platinum to the central nitrogen atom (Pt1-N1, 1.99 (2) Å) of the terpyridine ligand, which is slightly longer than to the other two outer nitrogen atoms (Pt1-N2 1.95 (2) Å, Pt1-N3 1.98 (2) Å). All the Pt-N distances are in the range generally observed in typical platinum terpyridine complexes [Chakraborty *et al.* (2005), Sakai *et al.* (2003)]. The bond angles (N1-Pt1-N2 82.7 (9)°, N1-Pt1-N3 80.6 (10)°, N2-Pt1-N3 163.2 (9)°, N1-Pt1-Cl5 179.1 (7)°) deviate from the idealized values of 90° and 180° as a consequence of the geometric constraints imposed by the terpyridine ligand.

### Experimental

The ligand 4'-p-tolyl-[2,2':6',2'']terpyridine was prepared by a literature method (Kronke, 1976), and bromination was carried out in the presence of N-bromosuccinimide and a small amount of benzoyl peroxide in CCl_4_. The title complex was prepared as previously reported (Jarosz *et al.*, 2008). The bipyridinium appended terpyridine ligand (0.56 g, 1 mmol), synthesized according to a previously reported procedure (Collin *et al.*, 1991), was mixed with Pt(DMSO)_2_Cl_2_ (0.47 g, 1.1 mmol) in 50 mL acetonitrile/chloroform (v/v, 2:1). The mixture was stirred at reflux for 30 h, and a saturated aqueous solution of sodium perchlorate was added after cooled to room temperature. The resulting orange precipitate was collected by filtration and washed with water and diethyl ether. Recrystallization by vapor diffusion of diethyl ether into a dichloromethane/acetonitrile (v/v, 1:1) mixture gave yellow crystals.

### Refinement

The H atoms were placed in calculated positions [aromatic C-H = 0.93Å and Uiso(H) = 1.2Ueq(C)]. Those of the methyl groups were rotated to fit the electron densidy [ C-H = 0.96Å and Uiso(H) = 1.5Ueq(C)]. The H atoms were included in the refinements in the riding-model approximation. The water H atoms were located in a difference Fourier map and fixed in these positions with distance restraints of  O-H = 0.85Å.

### Crystal data

**Table:**

[PtCl(C_32_H_24_N_5_)](ClO_4_)_2_·2C_2_H_3_N·1.5H_2_O	*F*(000) = 4024
*M**_r_* = 1017.14	*D*_x_ = 1.685 Mg m^−^^3^
Monoclinic, *P*2_1_/*n*	Mo *K*α radiation, λ = 0.71073 Å
Hall symbol: -P 2yn	Cell parameters from 3681 reflections
*a* = 13.3990 (15) Å	θ = 3.0–26.3°
*b* = 14.5018 (15) Å	µ = 3.76 mm^−^^1^
*c* = 41.279 (4) Å	*T* = 298 K
β = 90.390 (1)°	Block, yellow
*V* = 8020.8 (14) Å^3^	0.20 × 0.19 × 0.18 mm
*Z* = 8	

### Data collection

**Table:**

Bruker SMART diffractometer	14131 independent reflections
Radiation source: fine-focus sealed tube	5677 reflections with *I* > 2σ(*I*)
graphite	*R*_int_ = 0.143
φ and ω scans	θ_max_ = 25.0°, θ_min_ = 1.5°
Absorption correction: multi-scan (*SADABS*; Sheldrick, 1996)	*h* = −9→15
*T*_min_ = 0.520, *T*_max_ = 0.551	*k* = −17→17
41405 measured reflections	*l* = −49→49

### Refinement

**Table:**

Refinement on *F*^2^	Primary atom site location: structure-invariant direct methods
Least-squares matrix: full	Secondary atom site location: difference Fourier map
*R*[*F*^2^ > 2σ(*F*^2^)] = 0.114	Hydrogen site location: inferred from neighbouring sites
*wR*(*F*^2^) = 0.339	H-atom parameters constrained
*S* = 1.03	*w* = 1/[σ^2^(*F*_o_^2^) + (0.1592*P*)^2^] where *P* = (*F*_o_^2^ + 2*F*_c_^2^)/3
14131 reflections	(Δ/σ)_max_ = 0.001
1022 parameters	Δρ_max_ = 3.13 e Å^−^^3^
0 restraints	Δρ_min_ = −1.62 e Å^−^^3^

### Special details

**Table:**

Geometry. All esds (except the esd in the dihedral angle between two l.s. planes) are estimated using the full covariance matrix. The cell esds are taken into account individually in the estimation of esds in distances, angles and torsion angles; correlations between esds in cell parameters are only used when they are defined by crystal symmetry. An approximate (isotropic) treatment of cell esds is used for estimating esds involving l.s. planes.
Refinement. Refinement of F^2^ against ALL reflections. The weighted R-factor wR and goodness of fit S are based on F^2^, conventional R-factors R are based on F, with F set to zero for negative F^2^. The threshold expression of F^2^ > 2sigma(F^2^) is used only for calculating R-factors(gt) etc. and is not relevant to the choice of reflections for refinement. R-factors based on F^2^ are statistically about twice as large as those based on F, and R- factors based on ALL data will be even larger.

### Fractional atomic coordinates and isotropic or equivalent isotropic displacement parameters (Å^2^)

**Table:**

	*x*	*y*	*z*	*U*_iso_*/*U*_eq_	
Pt1	1.00170 (8)	0.10758 (6)	1.01667 (2)	0.0650 (4)	
Pt2	0.53663 (9)	0.11377 (6)	0.48389 (2)	0.0829 (4)	
Cl1	0.0671 (10)	0.0539 (12)	0.5808 (4)	0.164 (5)	
Cl2	0.5896 (12)	0.5208 (11)	0.7315 (5)	0.175 (5)	
Cl3	0.4122 (12)	−0.0009 (10)	0.7348 (4)	0.168 (5)	
Cl4	0.4248 (7)	0.0004 (8)	0.0713 (3)	0.125 (3)	
Cl5	1.0569 (4)	0.0586 (3)	1.06877 (12)	0.0551 (13)	
Cl6	0.5889 (6)	0.0758 (4)	0.43079 (13)	0.086 (2)	
N1	0.9535 (18)	0.1489 (12)	0.9734 (5)	0.067 (5)	
N2	1.1315 (15)	0.1115 (12)	0.9954 (5)	0.074 (6)	
N3	0.8571 (17)	0.1189 (13)	1.0260 (6)	0.079 (6)	
N4	0.755 (2)	0.3066 (18)	0.7641 (6)	0.099 (7)	
N5	0.688 (2)	0.0280 (17)	0.6237 (6)	0.107 (8)	
N6	0.496 (2)	0.1483 (14)	0.5265 (6)	0.085 (7)	
N7	0.670 (2)	0.1392 (14)	0.5035 (6)	0.090 (7)	
N8	0.3955 (18)	0.0981 (13)	0.4785 (5)	0.081 (6)	
N9	0.276 (2)	0.2922 (18)	0.7373 (6)	0.102 (8)	
N10	0.210 (3)	0.0023 (18)	0.8747 (6)	0.112 (8)	
N11	1.044 (3)	0.227 (3)	0.4477 (10)	0.171 (15)	
N12	0.745 (5)	0.504 (4)	0.6440 (16)	0.27 (3)	
N13	0.497 (3)	0.381 (3)	0.8142 (9)	0.154 (13)	
N14	0.208 (3)	0.286 (3)	0.8722 (10)	0.162 (14)	
O1	0.089 (3)	0.148 (3)	0.5762 (11)	0.27 (2)	
O2	0.005 (4)	0.043 (5)	0.6071 (13)	0.47 (5)	
O3	0.156 (3)	0.006 (4)	0.5856 (10)	0.35 (3)	
O4	0.020 (5)	0.021 (5)	0.5527 (16)	0.38 (3)	
O5	0.574 (5)	0.581 (5)	0.7056 (18)	0.39 (3)	
O6	0.686 (5)	0.484 (4)	0.7293 (18)	0.48 (5)	
O7	0.520 (5)	0.449 (6)	0.7302 (16)	0.50 (6)	
O8	0.580 (7)	0.568 (7)	0.761 (2)	0.48 (5)	
O9	0.465 (4)	−0.066 (4)	0.7172 (13)	0.31 (2)	
O10	0.313 (3)	−0.027 (3)	0.7382 (11)	0.33 (3)	
O11	0.421 (3)	0.087 (3)	0.7216 (10)	0.252 (18)	
O12	0.457 (7)	0.011 (5)	0.765 (2)	0.65 (9)	
O13	0.466 (2)	−0.027 (3)	0.0417 (7)	0.214 (16)	
O14	0.413 (4)	0.096 (3)	0.0707 (12)	0.32 (3)	
O15	0.489 (3)	−0.024 (3)	0.0965 (10)	0.239 (16)	
O16	0.3319 (18)	−0.041 (2)	0.0752 (9)	0.224 (15)	
O17	0.021 (3)	0.345 (4)	0.6881 (10)	0.33 (3)	
H17C	0.0247	0.3997	0.6950	0.400*	
H17D	0.0128	0.3452	0.6677	0.400*	
O18	0.497 (4)	0.216 (3)	0.1285 (13)	0.38 (3)	
H18C	0.5250	0.2574	0.1173	0.450*	
H18D	0.4697	0.1775	0.1156	0.450*	
O19	0.917 (6)	0.590 (5)	0.6892 (18)	0.52 (5)	
H19C	0.9489	0.5565	0.7027	0.624*	
H19D	0.8730	0.5564	0.6798	0.624*	
C1	1.0300 (19)	0.1675 (15)	0.9510 (6)	0.065 (6)	
C2	0.995 (2)	0.1954 (15)	0.9202 (6)	0.074 (7)	
H2	1.0409	0.2039	0.9035	0.088*	
C3	0.893 (2)	0.2106 (16)	0.9142 (6)	0.073 (7)	
C4	0.824 (2)	0.1924 (16)	0.9392 (7)	0.078 (7)	
H4	0.7568	0.1989	0.9346	0.093*	
C5	0.853 (2)	0.1650 (16)	0.9702 (7)	0.070 (7)	
C6	1.133 (2)	0.1401 (15)	0.9633 (7)	0.071 (7)	
C7	1.211 (2)	0.1420 (16)	0.9419 (7)	0.079 (7)	
H7	1.2028	0.1576	0.9202	0.095*	
C8	1.304 (2)	0.1183 (16)	0.9557 (7)	0.084 (8)	
H8	1.3615	0.1193	0.9431	0.101*	
C9	1.309 (2)	0.0935 (16)	0.9882 (7)	0.085 (8)	
H9	1.3710	0.0782	0.9973	0.102*	
C10	1.225 (2)	0.0911 (16)	1.0073 (7)	0.082 (8)	
H10	1.2320	0.0752	1.0290	0.098*	
C11	0.798 (2)	0.1477 (17)	0.9999 (7)	0.078 (7)	
C12	0.695 (2)	0.1630 (16)	1.0016 (7)	0.079 (7)	
H12	0.6595	0.1810	0.9832	0.095*	
C13	0.646 (2)	0.1513 (17)	1.0309 (7)	0.083 (7)	
H13	0.5782	0.1638	1.0325	0.100*	
C14	0.699 (2)	0.1210 (16)	1.0576 (7)	0.081 (7)	
H14	0.6678	0.1090	1.0771	0.097*	
C15	0.802 (2)	0.1087 (16)	1.0543 (7)	0.082 (7)	
H15	0.8372	0.0921	1.0729	0.099*	
C16	0.857 (2)	0.2462 (18)	0.8811 (6)	0.084 (7)	
C17	0.928 (2)	0.2789 (18)	0.8595 (7)	0.089 (8)	
H17	0.9955	0.2748	0.8646	0.107*	
C18	0.898 (2)	0.318 (2)	0.8302 (7)	0.100 (9)	
H18	0.9451	0.3399	0.8158	0.120*	
C19	0.797 (3)	0.324 (2)	0.8226 (7)	0.096 (8)	
C20	0.726 (2)	0.291 (2)	0.8442 (8)	0.101 (9)	
H20	0.6584	0.2954	0.8391	0.121*	
C21	0.756 (2)	0.2523 (19)	0.8734 (7)	0.093 (8)	
H21	0.7088	0.2303	0.8879	0.112*	
C22	0.761 (2)	0.374 (2)	0.7931 (7)	0.101 (9)	
H22A	0.8063	0.4245	0.7881	0.121*	
H22B	0.6955	0.4006	0.7971	0.121*	
C23	0.823 (2)	0.310 (2)	0.7389 (7)	0.104 (9)	
H23	0.8770	0.3500	0.7392	0.125*	
C24	0.806 (2)	0.249 (2)	0.7132 (7)	0.104 (9)	
H24	0.8532	0.2475	0.6968	0.125*	
C25	0.724 (3)	0.192 (2)	0.7104 (7)	0.100 (9)	
C26	0.661 (2)	0.193 (2)	0.7368 (7)	0.103 (9)	
H26	0.6086	0.1511	0.7369	0.124*	
C27	0.672 (2)	0.251 (2)	0.7630 (7)	0.101 (9)	
H27	0.6252	0.2521	0.7795	0.122*	
C28	0.781 (3)	0.048 (2)	0.6364 (8)	0.108 (10)	
H28	0.8375	0.0220	0.6271	0.129*	
C29	0.790 (3)	0.106 (2)	0.6631 (8)	0.110 (10)	
H29	0.8527	0.1270	0.6690	0.131*	
C30	0.709 (3)	0.133 (2)	0.6809 (8)	0.103 (9)	
C31	0.617 (3)	0.103 (2)	0.6700 (7)	0.101 (9)	
H31	0.5609	0.1150	0.6822	0.121*	
C32	0.606 (3)	0.054 (2)	0.6414 (8)	0.103 (9)	
H32	0.5428	0.0395	0.6340	0.124*	
C33	0.561 (3)	0.170 (2)	0.5499 (8)	0.093 (9)	
C34	0.530 (3)	0.2014 (17)	0.5799 (7)	0.092 (9)	
H34	0.5770	0.2237	0.5946	0.110*	
C35	0.431 (3)	0.2003 (17)	0.5884 (7)	0.085 (8)	
C36	0.364 (2)	0.1746 (17)	0.5633 (7)	0.089 (8)	
H36	0.2960	0.1782	0.5673	0.107*	
C37	0.395 (3)	0.1446 (17)	0.5334 (7)	0.085 (8)	
C38	0.667 (3)	0.171 (2)	0.5354 (8)	0.098 (9)	
C39	0.753 (3)	0.1968 (19)	0.5525 (8)	0.099 (9)	
H39	0.7468	0.2157	0.5739	0.119*	
C40	0.848 (3)	0.1950 (18)	0.5382 (8)	0.099 (9)	
H40	0.9058	0.2091	0.5498	0.118*	
C41	0.850 (3)	0.170 (2)	0.5055 (9)	0.104 (9)	
H41	0.9094	0.1742	0.4942	0.125*	
C42	0.764 (3)	0.1401 (19)	0.4894 (8)	0.099 (9)	
H42	0.7696	0.1195	0.4682	0.119*	
C43	0.334 (3)	0.1154 (17)	0.5051 (7)	0.085 (8)	
C44	0.231 (3)	0.1084 (18)	0.5069 (7)	0.092 (9)	
H44	0.1964	0.1257	0.5253	0.110*	
C45	0.184 (2)	0.0736 (17)	0.4792 (7)	0.090 (8)	
H45	0.1164	0.0582	0.4803	0.108*	
C46	0.234 (3)	0.0613 (18)	0.4504 (7)	0.091 (8)	
H46	0.1996	0.0486	0.4312	0.110*	
C47	0.338 (3)	0.0689 (17)	0.4511 (7)	0.090 (8)	
H47	0.3728	0.0537	0.4323	0.108*	
C48	0.397 (3)	0.2339 (19)	0.6212 (7)	0.092 (8)	
C49	0.461 (2)	0.2842 (19)	0.6410 (7)	0.097 (9)	
H49	0.5269	0.2936	0.6348	0.117*	
C50	0.427 (3)	0.3202 (19)	0.6701 (7)	0.100 (9)	
H50	0.4699	0.3539	0.6834	0.119*	
C51	0.329 (3)	0.306 (2)	0.6794 (7)	0.100 (9)	
C52	0.264 (3)	0.256 (2)	0.6595 (7)	0.103 (9)	
H52	0.1986	0.2463	0.6657	0.123*	
C53	0.299 (3)	0.2197 (19)	0.6305 (7)	0.097 (9)	
H53	0.2555	0.1861	0.6172	0.116*	
C54	0.290 (2)	0.3566 (19)	0.7086 (7)	0.103 (9)	
H54A	0.2264	0.3851	0.7032	0.123*	
H54B	0.3363	0.4052	0.7145	0.123*	
C55	0.350 (3)	0.284 (2)	0.7604 (9)	0.116 (11)	
H55	0.4085	0.3171	0.7583	0.139*	
C56	0.337 (3)	0.226 (2)	0.7869 (8)	0.111 (10)	
H56	0.3873	0.2223	0.8025	0.133*	
C57	0.250 (3)	0.174 (2)	0.7905 (8)	0.109 (10)	
C58	0.175 (3)	0.190 (2)	0.7679 (8)	0.110 (10)	
H58	0.1137	0.1599	0.7708	0.132*	
C59	0.186 (3)	0.248 (2)	0.7405 (7)	0.107 (10)	
H59	0.1343	0.2565	0.7255	0.128*	
C60	0.128 (3)	0.038 (2)	0.8590 (8)	0.117 (11)	
H60	0.0645	0.0246	0.8667	0.140*	
C61	0.139 (3)	0.096 (2)	0.8310 (7)	0.111 (10)	
H61	0.0830	0.1197	0.8203	0.133*	
C62	0.236 (3)	0.115 (2)	0.8200 (8)	0.113 (10)	
C63	0.320 (3)	0.078 (2)	0.8361 (8)	0.113 (10)	
H63	0.3843	0.0925	0.8297	0.135*	
C64	0.302 (3)	0.020 (2)	0.8615 (8)	0.114 (10)	
H64	0.3567	−0.0097	0.8708	0.137*	
C65	1.010 (4)	0.223 (3)	0.4218 (14)	0.17 (2)	
C66	0.955 (4)	0.225 (3)	0.3905 (11)	0.20 (2)	
H66A	0.8852	0.2360	0.3945	0.303*	
H66B	0.9624	0.1672	0.3795	0.303*	
H66C	0.9805	0.2737	0.3771	0.303*	
C67	0.725 (6)	0.425 (5)	0.6406 (18)	0.24 (3)	
C68	0.720 (3)	0.329 (3)	0.6295 (9)	0.154 (14)	
H68A	0.6862	0.3256	0.6090	0.231*	
H68B	0.6850	0.2923	0.6451	0.231*	
H68C	0.7868	0.3049	0.6272	0.231*	
C69	0.459 (4)	0.453 (4)	0.8142 (11)	0.161 (17)	
C70	0.409 (4)	0.544 (3)	0.8068 (11)	0.20 (2)	
H70A	0.4479	0.5768	0.7912	0.301*	
H70B	0.4045	0.5794	0.8263	0.301*	
H70C	0.3436	0.5324	0.7983	0.301*	
C71	0.198 (4)	0.359 (4)	0.8609 (14)	0.19 (2)	
C72	0.195 (4)	0.461 (4)	0.8581 (12)	0.21 (2)	
H72A	0.1360	0.4840	0.8684	0.321*	
H72B	0.1940	0.4781	0.8356	0.321*	
H72C	0.2529	0.4871	0.8684	0.321*	

### Atomic displacement parameters (Å^2^)

**Table:**

	*U*^11^	*U*^22^	*U*^33^	*U*^12^	*U*^13^	*U*^23^
Pt1	0.0891 (8)	0.0383 (5)	0.0674 (6)	−0.0043 (5)	−0.0096 (5)	−0.0055 (4)
Pt2	0.1299 (11)	0.0437 (6)	0.0754 (8)	0.0258 (6)	0.0100 (6)	0.0125 (5)
Cl1	0.132 (10)	0.190 (13)	0.169 (12)	−0.009 (9)	−0.016 (8)	0.050 (10)
Cl2	0.160 (12)	0.157 (12)	0.209 (16)	0.004 (10)	−0.036 (11)	0.013 (11)
Cl3	0.188 (14)	0.139 (11)	0.178 (13)	−0.007 (10)	0.054 (10)	0.036 (9)
Cl4	0.089 (7)	0.133 (8)	0.152 (9)	−0.007 (6)	−0.024 (6)	−0.002 (7)
Cl5	0.077 (4)	0.035 (3)	0.053 (3)	0.001 (2)	−0.013 (3)	−0.008 (2)
Cl6	0.140 (6)	0.068 (4)	0.051 (4)	0.052 (4)	0.017 (4)	0.000 (3)
N1	0.072 (16)	0.044 (11)	0.085 (16)	−0.015 (10)	−0.016 (13)	−0.005 (9)
N2	0.075 (15)	0.054 (12)	0.092 (17)	−0.008 (11)	−0.032 (12)	−0.001 (11)
N3	0.094 (17)	0.058 (13)	0.086 (16)	−0.001 (11)	−0.012 (14)	0.000 (11)
N4	0.11 (2)	0.10 (2)	0.085 (18)	−0.007 (16)	0.001 (15)	−0.005 (15)
N5	0.12 (2)	0.101 (19)	0.096 (19)	0.002 (17)	0.004 (18)	−0.008 (14)
N6	0.12 (2)	0.057 (13)	0.079 (17)	0.020 (13)	−0.003 (16)	0.020 (11)
N7	0.11 (2)	0.077 (15)	0.079 (18)	0.017 (13)	0.006 (15)	0.001 (12)
N8	0.14 (2)	0.055 (12)	0.051 (13)	0.025 (12)	0.021 (13)	0.018 (10)
N9	0.13 (2)	0.088 (18)	0.082 (18)	−0.006 (17)	0.003 (17)	0.002 (14)
N10	0.15 (3)	0.10 (2)	0.092 (19)	−0.017 (19)	−0.014 (19)	0.015 (15)
N11	0.19 (4)	0.13 (3)	0.19 (4)	0.02 (2)	−0.04 (3)	−0.01 (3)
N12	0.31 (7)	0.18 (5)	0.34 (7)	0.00 (5)	−0.01 (5)	0.02 (5)
N13	0.16 (3)	0.14 (3)	0.16 (3)	0.01 (2)	−0.01 (2)	0.02 (3)
N14	0.19 (4)	0.15 (3)	0.15 (3)	0.00 (3)	0.00 (3)	−0.01 (3)
O1	0.23 (4)	0.20 (4)	0.37 (6)	0.02 (3)	0.05 (4)	0.08 (4)
O2	0.31 (6)	0.75 (12)	0.34 (7)	−0.02 (7)	0.23 (6)	0.13 (7)
O3	0.22 (4)	0.50 (8)	0.33 (5)	0.18 (5)	−0.04 (4)	0.13 (5)
O4	0.38 (8)	0.38 (9)	0.38 (8)	−0.01 (6)	−0.01 (7)	0.00 (6)
O5	0.39 (9)	0.39 (9)	0.39 (9)	0.00 (7)	0.00 (7)	0.02 (7)
O6	0.35 (7)	0.35 (8)	0.73 (12)	0.26 (7)	0.04 (8)	0.17 (7)
O7	0.44 (9)	0.70 (13)	0.38 (8)	−0.38 (10)	−0.07 (6)	0.26 (9)
O8	0.47 (13)	0.48 (12)	0.48 (12)	0.00 (10)	0.00 (10)	0.00 (10)
O9	0.32 (6)	0.31 (6)	0.32 (6)	0.01 (5)	0.01 (5)	0.00 (5)
O10	0.16 (3)	0.43 (7)	0.39 (6)	−0.06 (4)	0.11 (4)	0.18 (5)
O11	0.25 (4)	0.21 (4)	0.30 (5)	0.05 (3)	0.09 (4)	0.04 (3)
O12	0.93 (18)	0.37 (9)	0.63 (12)	0.12 (10)	−0.59 (14)	0.11 (9)
O13	0.16 (3)	0.33 (4)	0.15 (3)	0.06 (3)	0.07 (2)	0.04 (3)
O14	0.38 (6)	0.17 (3)	0.42 (7)	−0.06 (4)	0.18 (5)	−0.16 (4)
O15	0.24 (4)	0.25 (5)	0.23 (4)	0.01 (3)	−0.01 (3)	0.00 (3)
O16	0.087 (19)	0.20 (3)	0.38 (5)	−0.08 (2)	0.01 (2)	0.01 (3)
O17	0.16 (3)	0.58 (9)	0.26 (5)	−0.03 (4)	−0.01 (3)	0.08 (5)
O18	0.49 (8)	0.12 (3)	0.52 (8)	0.04 (4)	−0.09 (6)	0.02 (4)
O19	0.52 (13)	0.52 (12)	0.52 (11)	0.00 (9)	0.00 (9)	−0.01 (9)
C1	0.077 (19)	0.043 (13)	0.077 (18)	0.003 (12)	−0.016 (15)	−0.004 (12)
C2	0.08 (2)	0.060 (16)	0.08 (2)	−0.009 (13)	−0.011 (14)	−0.005 (13)
C3	0.08 (2)	0.065 (16)	0.079 (19)	−0.009 (13)	−0.010 (15)	−0.004 (13)
C4	0.08 (2)	0.065 (17)	0.08 (2)	−0.009 (13)	−0.014 (16)	0.002 (14)
C5	0.08 (2)	0.050 (14)	0.08 (2)	−0.008 (13)	−0.009 (16)	−0.005 (13)
C6	0.07 (2)	0.057 (15)	0.09 (2)	−0.019 (12)	−0.025 (17)	−0.018 (13)
C7	0.08 (2)	0.069 (17)	0.09 (2)	−0.005 (14)	−0.015 (18)	−0.010 (13)
C8	0.09 (2)	0.072 (18)	0.09 (2)	−0.003 (15)	−0.015 (16)	−0.007 (15)
C9	0.08 (2)	0.071 (18)	0.10 (2)	0.003 (14)	−0.023 (17)	0.002 (16)
C10	0.08 (2)	0.068 (18)	0.09 (2)	0.001 (14)	−0.013 (18)	−0.007 (14)
C11	0.08 (2)	0.061 (16)	0.09 (2)	−0.012 (14)	−0.015 (18)	−0.002 (14)
C12	0.08 (2)	0.062 (16)	0.09 (2)	−0.003 (15)	−0.006 (16)	0.009 (14)
C13	0.09 (2)	0.067 (17)	0.09 (2)	−0.004 (15)	−0.005 (18)	−0.004 (15)
C14	0.09 (2)	0.065 (17)	0.09 (2)	−0.004 (15)	−0.004 (16)	0.003 (14)
C15	0.09 (2)	0.067 (17)	0.09 (2)	−0.007 (16)	−0.015 (16)	0.002 (14)
C16	0.09 (2)	0.082 (19)	0.08 (2)	−0.003 (16)	−0.013 (17)	0.011 (15)
C17	0.09 (2)	0.09 (2)	0.08 (2)	−0.005 (16)	−0.010 (17)	0.012 (15)
C18	0.10 (3)	0.11 (2)	0.09 (2)	−0.006 (19)	−0.006 (18)	0.012 (18)
C19	0.10 (3)	0.10 (2)	0.08 (2)	−0.006 (19)	−0.007 (19)	0.004 (18)
C20	0.10 (2)	0.11 (2)	0.09 (2)	−0.004 (18)	−0.002 (19)	0.007 (18)
C21	0.09 (2)	0.10 (2)	0.09 (2)	−0.010 (17)	−0.004 (17)	0.013 (17)
C22	0.11 (2)	0.10 (2)	0.09 (2)	−0.005 (17)	−0.003 (17)	−0.001 (19)
C23	0.11 (3)	0.11 (3)	0.09 (2)	−0.014 (19)	−0.003 (19)	−0.013 (19)
C24	0.11 (3)	0.11 (2)	0.09 (2)	−0.01 (2)	0.005 (18)	−0.016 (19)
C25	0.11 (3)	0.10 (2)	0.09 (2)	−0.010 (19)	0.004 (19)	−0.008 (18)
C26	0.11 (3)	0.11 (2)	0.09 (2)	−0.013 (19)	0.005 (19)	−0.009 (19)
C27	0.11 (3)	0.11 (2)	0.09 (2)	−0.01 (2)	0.004 (18)	−0.007 (19)
C28	0.11 (3)	0.11 (3)	0.10 (3)	0.00 (2)	0.01 (2)	−0.012 (19)
C29	0.12 (3)	0.11 (3)	0.10 (2)	−0.01 (2)	0.00 (2)	−0.01 (2)
C30	0.11 (3)	0.10 (2)	0.09 (2)	−0.008 (19)	0.01 (2)	−0.009 (18)
C31	0.11 (3)	0.10 (2)	0.09 (2)	0.00 (2)	0.005 (19)	−0.008 (18)
C32	0.12 (3)	0.10 (2)	0.10 (2)	−0.01 (2)	0.01 (2)	−0.003 (18)
C33	0.13 (3)	0.075 (19)	0.08 (2)	0.013 (18)	0.00 (2)	0.013 (16)
C34	0.12 (3)	0.072 (19)	0.08 (2)	0.009 (17)	0.004 (18)	0.008 (15)
C35	0.12 (3)	0.064 (17)	0.07 (2)	0.009 (16)	−0.004 (19)	0.006 (14)
C36	0.12 (2)	0.066 (17)	0.08 (2)	0.015 (17)	0.001 (18)	0.013 (15)
C37	0.12 (3)	0.064 (17)	0.07 (2)	0.024 (16)	−0.002 (19)	0.014 (14)
C38	0.13 (3)	0.08 (2)	0.09 (3)	0.022 (19)	0.00 (2)	0.011 (17)
C39	0.13 (3)	0.08 (2)	0.09 (2)	0.015 (19)	0.00 (2)	0.006 (16)
C40	0.12 (3)	0.08 (2)	0.10 (3)	0.015 (17)	0.00 (2)	0.007 (17)
C41	0.12 (3)	0.08 (2)	0.11 (3)	0.02 (2)	0.00 (2)	0.005 (19)
C42	0.12 (3)	0.08 (2)	0.10 (2)	0.020 (18)	0.00 (2)	0.007 (16)
C43	0.12 (3)	0.063 (17)	0.07 (2)	0.021 (18)	0.001 (18)	0.011 (14)
C44	0.13 (3)	0.074 (18)	0.08 (2)	0.025 (19)	0.003 (18)	0.007 (15)
C45	0.12 (2)	0.068 (17)	0.08 (2)	0.021 (16)	−0.004 (19)	0.012 (15)
C46	0.13 (3)	0.073 (18)	0.07 (2)	0.018 (18)	−0.007 (19)	0.008 (14)
C47	0.13 (3)	0.066 (17)	0.08 (2)	0.025 (17)	0.005 (19)	0.011 (14)
C48	0.12 (3)	0.072 (19)	0.08 (2)	0.002 (18)	−0.004 (19)	0.008 (15)
C49	0.12 (3)	0.08 (2)	0.09 (2)	0.000 (18)	0.001 (19)	−0.007 (16)
C50	0.13 (3)	0.08 (2)	0.08 (2)	−0.008 (19)	0.000 (19)	0.007 (17)
C51	0.13 (3)	0.09 (2)	0.08 (2)	−0.01 (2)	0.00 (2)	0.001 (17)
C52	0.13 (3)	0.09 (2)	0.09 (2)	−0.011 (19)	0.00 (2)	0.002 (18)
C53	0.12 (3)	0.08 (2)	0.08 (2)	−0.004 (18)	0.007 (19)	0.000 (16)
C54	0.13 (3)	0.09 (2)	0.09 (2)	−0.008 (18)	0.003 (19)	−0.001 (17)
C55	0.14 (3)	0.10 (3)	0.10 (3)	−0.01 (2)	0.00 (2)	0.01 (2)
C56	0.14 (3)	0.10 (2)	0.09 (2)	−0.01 (2)	0.00 (2)	0.007 (18)
C57	0.14 (3)	0.09 (2)	0.09 (2)	−0.01 (2)	0.00 (2)	0.008 (19)
C58	0.14 (3)	0.10 (2)	0.10 (3)	−0.01 (2)	0.00 (2)	0.006 (19)
C59	0.14 (3)	0.09 (2)	0.09 (2)	−0.01 (2)	0.00 (2)	0.004 (18)
C60	0.15 (3)	0.10 (3)	0.10 (3)	−0.02 (2)	0.00 (2)	0.01 (2)
C61	0.14 (3)	0.10 (2)	0.09 (2)	−0.01 (2)	0.00 (2)	0.008 (18)
C62	0.14 (3)	0.10 (2)	0.09 (2)	−0.02 (2)	0.00 (2)	0.010 (19)
C63	0.14 (3)	0.10 (2)	0.10 (2)	−0.01 (2)	0.00 (2)	0.011 (19)
C64	0.14 (3)	0.10 (3)	0.10 (2)	0.00 (2)	−0.01 (2)	0.016 (19)
C65	0.19 (5)	0.14 (4)	0.18 (5)	0.02 (3)	−0.07 (4)	0.01 (4)
C66	0.21 (5)	0.18 (5)	0.21 (5)	0.01 (4)	−0.05 (4)	−0.01 (4)
C67	0.27 (7)	0.16 (6)	0.29 (7)	−0.01 (6)	−0.02 (5)	−0.01 (6)
C68	0.18 (4)	0.12 (3)	0.17 (4)	−0.01 (3)	−0.01 (3)	0.01 (3)
C69	0.17 (5)	0.15 (5)	0.16 (4)	0.01 (4)	0.00 (3)	0.01 (4)
C70	0.21 (5)	0.18 (5)	0.21 (5)	0.03 (4)	0.00 (4)	0.00 (4)
C71	0.20 (5)	0.16 (5)	0.20 (6)	0.02 (5)	0.02 (4)	0.00 (5)
C72	0.21 (5)	0.20 (6)	0.23 (6)	0.02 (5)	−0.01 (4)	0.00 (5)

### Geometric parameters (Å, °)

**Table:**

Pt1—N2	1.95 (2)	C21—H21	0.9300
Pt1—N3	1.98 (2)	C22—H22A	0.9700
Pt1—N1	1.99 (2)	C22—H22B	0.9700
Pt1—Cl5	2.378 (5)	C23—C24	1.39 (4)
Pt2—N6	1.91 (2)	C23—H23	0.9300
Pt2—N8	1.92 (2)	C24—C25	1.38 (4)
Pt2—N7	1.99 (3)	C24—H24	0.9300
Pt2—Cl6	2.370 (5)	C25—C26	1.38 (3)
Cl1—O2	1.38 (4)	C25—C30	1.50 (4)
Cl1—O3	1.40 (4)	C26—C27	1.38 (3)
Cl1—O4	1.40 (7)	C26—H26	0.9300
Cl1—O1	1.40 (4)	C27—H27	0.9300
Cl2—O5	1.40 (7)	C28—C29	1.39 (4)
Cl2—O8	1.40 (9)	C28—H28	0.9300
Cl2—O7	1.40 (6)	C29—C30	1.37 (4)
Cl2—O6	1.40 (5)	C29—H29	0.9300
Cl3—O11	1.39 (4)	C30—C31	1.38 (4)
Cl3—O9	1.39 (5)	C31—C32	1.38 (4)
Cl3—O10	1.39 (4)	C31—H31	0.9300
Cl3—O12	1.40 (6)	C32—H32	0.9300
Cl4—O15	1.39 (4)	C33—C34	1.38 (4)
Cl4—O16	1.39 (2)	C33—C38	1.54 (4)
Cl4—O14	1.40 (4)	C34—C35	1.38 (4)
Cl4—O13	1.40 (3)	C34—H34	0.9300
N1—C5	1.38 (3)	C35—C36	1.41 (4)
N1—C1	1.41 (3)	C35—C48	1.51 (4)
N2—C10	1.38 (3)	C36—C37	1.38 (4)
N2—C6	1.39 (3)	C36—H36	0.9300
N3—C15	1.40 (3)	C37—C43	1.48 (4)
N3—C11	1.40 (3)	C38—C39	1.39 (4)
N4—C27	1.37 (3)	C39—C40	1.41 (4)
N4—C23	1.39 (3)	C39—H39	0.9300
N4—C22	1.55 (3)	C40—C41	1.40 (4)
N5—C32	1.37 (3)	C40—H40	0.9300
N5—C28	1.38 (4)	C41—C42	1.40 (4)
N6—C33	1.34 (3)	C41—H41	0.9300
N6—C37	1.38 (3)	C42—H42	0.9300
N7—C42	1.39 (4)	C43—C44	1.38 (4)
N7—C38	1.40 (3)	C44—C45	1.40 (4)
N8—C43	1.40 (3)	C44—H44	0.9300
N8—C47	1.43 (3)	C45—C46	1.38 (3)
N9—C55	1.37 (4)	C45—H45	0.9300
N9—C59	1.38 (4)	C46—C47	1.40 (4)
N9—C54	1.52 (3)	C46—H46	0.9300
N10—C60	1.37 (4)	C47—H47	0.9300
N10—C64	1.38 (4)	C48—C53	1.39 (4)
N11—C65	1.16 (5)	C48—C49	1.39 (4)
N12—C67	1.18 (6)	C49—C50	1.39 (3)
N13—C69	1.16 (5)	C49—H49	0.9300
N14—C71	1.16 (5)	C50—C51	1.39 (4)
O17—H17C	0.8499	C50—H50	0.9300
O17—H17D	0.8499	C51—C52	1.39 (4)
O18—H18C	0.8500	C51—C54	1.51 (4)
O18—H18D	0.8502	C52—C53	1.39 (3)
O19—H19C	0.8501	C52—H52	0.9300
O19—H19D	0.8501	C53—H53	0.9300
C1—C2	1.41 (3)	C54—H54A	0.9700
C1—C6	1.52 (3)	C54—H54B	0.9700
C2—C3	1.41 (3)	C55—C56	1.39 (4)
C2—H2	0.9300	C55—H55	0.9300
C3—C4	1.41 (3)	C56—C57	1.40 (4)
C3—C16	1.54 (3)	C56—H56	0.9300
C4—C5	1.39 (3)	C57—C58	1.39 (4)
C4—H4	0.9300	C57—C62	1.51 (4)
C5—C11	1.46 (3)	C58—C59	1.42 (4)
C6—C7	1.37 (3)	C58—H58	0.9300
C7—C8	1.41 (3)	C59—H59	0.9300
C7—H7	0.9300	C60—C61	1.44 (4)
C8—C9	1.39 (3)	C60—H60	0.9300
C8—H8	0.9300	C61—C62	1.41 (4)
C9—C10	1.38 (3)	C61—H61	0.9300
C9—H9	0.9300	C62—C63	1.40 (4)
C10—H10	0.9300	C63—C64	1.37 (4)
C11—C12	1.40 (3)	C63—H63	0.9300
C12—C13	1.39 (3)	C64—H64	0.9300
C12—H12	0.9300	C65—C66	1.49 (5)
C13—C14	1.38 (3)	C66—H66A	0.9600
C13—H13	0.9300	C66—H66B	0.9600
C14—C15	1.39 (3)	C66—H66C	0.9600
C14—H14	0.9300	C67—C68	1.48 (7)
C15—H15	0.9300	C68—H68A	0.9600
C16—C17	1.39 (3)	C68—H68B	0.9600
C16—C21	1.39 (4)	C68—H68C	0.9600
C17—C18	1.39 (3)	C69—C70	1.50 (6)
C17—H17	0.9300	C70—H70A	0.9600
C18—C19	1.39 (4)	C70—H70B	0.9600
C18—H18	0.9300	C70—H70C	0.9600
C19—C20	1.39 (4)	C71—C72	1.49 (6)
C19—C22	1.50 (4)	C72—H72A	0.9600
C20—C21	1.39 (3)	C72—H72B	0.9600
C20—H20	0.9300	C72—H72C	0.9600
			
N2—Pt1—N3	163.2 (9)	N4—C27—C26	118 (3)
N2—Pt1—N1	82.7 (9)	N4—C27—H27	121.1
N3—Pt1—N1	80.6 (10)	C26—C27—H27	121.1
N2—Pt1—Cl5	98.2 (6)	N5—C28—C29	120 (3)
N3—Pt1—Cl5	98.5 (7)	N5—C28—H28	120.1
N1—Pt1—Cl5	179.1 (7)	C29—C28—H28	120.1
N6—Pt2—N8	81.2 (11)	C30—C29—C28	122 (3)
N6—Pt2—N7	80.8 (12)	C30—C29—H29	118.8
N8—Pt2—N7	162.0 (10)	C28—C29—H29	118.8
N6—Pt2—Cl6	178.2 (7)	C29—C30—C31	116 (3)
N8—Pt2—Cl6	99.4 (6)	C29—C30—C25	120 (3)
N7—Pt2—Cl6	98.6 (8)	C31—C30—C25	124 (3)
O2—Cl1—O3	110 (3)	C32—C31—C30	122 (3)
O2—Cl1—O4	110 (4)	C32—C31—H31	118.9
O3—Cl1—O4	109 (4)	C30—C31—H31	118.9
O2—Cl1—O1	110 (4)	N5—C32—C31	121 (3)
O3—Cl1—O1	109 (3)	N5—C32—H32	119.3
O4—Cl1—O1	109 (3)	C31—C32—H32	119.3
O5—Cl2—O8	110 (5)	N6—C33—C34	121 (3)
O5—Cl2—O7	110 (4)	N6—C33—C38	109 (3)
O8—Cl2—O7	109 (5)	C34—C33—C38	129 (3)
O5—Cl2—O6	109 (4)	C35—C34—C33	122 (3)
O8—Cl2—O6	109 (5)	C35—C34—H34	119.2
O7—Cl2—O6	109 (5)	C33—C34—H34	119.2
O11—Cl3—O9	112 (3)	C34—C35—C36	115 (3)
O11—Cl3—O10	112 (3)	C34—C35—C48	121 (3)
O9—Cl3—O10	111 (3)	C36—C35—C48	123 (3)
O11—Cl3—O12	102 (4)	C37—C36—C35	123 (3)
O9—Cl3—O12	109 (4)	C37—C36—H36	118.3
O10—Cl3—O12	111 (5)	C35—C36—H36	118.3
O15—Cl4—O16	111 (2)	C36—C37—N6	118 (3)
O15—Cl4—O14	109 (3)	C36—C37—C43	129 (3)
O16—Cl4—O14	109 (2)	N6—C37—C43	112 (3)
O15—Cl4—O13	110 (2)	C39—C38—N7	122 (3)
O16—Cl4—O13	110 (2)	C39—C38—C33	124 (3)
O14—Cl4—O13	108 (2)	N7—C38—C33	113 (3)
C5—N1—C1	128 (2)	C38—C39—C40	122 (3)
C5—N1—Pt1	116.9 (17)	C38—C39—H39	119.0
C1—N1—Pt1	114.5 (16)	C40—C39—H39	119.0
C10—N2—C6	113 (2)	C41—C40—C39	115 (3)
C10—N2—Pt1	130 (2)	C41—C40—H40	122.3
C6—N2—Pt1	117.2 (17)	C39—C40—H40	122.3
C15—N3—C11	112 (2)	C42—C41—C40	121 (3)
C15—N3—Pt1	133 (2)	C42—C41—H41	119.3
C11—N3—Pt1	115.2 (19)	C40—C41—H41	119.3
C27—N4—C23	122 (3)	N7—C42—C41	123 (3)
C27—N4—C22	116 (3)	N7—C42—H42	118.3
C23—N4—C22	122 (3)	C41—C42—H42	118.3
C32—N5—C28	117 (3)	C44—C43—N8	128 (3)
C33—N6—C37	120 (3)	C44—C43—C37	121 (3)
C33—N6—Pt2	122 (2)	N8—C43—C37	110 (3)
C37—N6—Pt2	118 (2)	C43—C44—C45	116 (3)
C42—N7—C38	115 (3)	C43—C44—H44	122.2
C42—N7—Pt2	130 (2)	C45—C44—H44	122.2
C38—N7—Pt2	114 (2)	C46—C45—C44	122 (3)
C43—N8—C47	111 (3)	C46—C45—H45	119.1
C43—N8—Pt2	118 (2)	C44—C45—H45	119.1
C47—N8—Pt2	130.6 (18)	C45—C46—C47	118 (3)
C55—N9—C59	121 (3)	C45—C46—H46	121.2
C55—N9—C54	121 (3)	C47—C46—H46	121.2
C59—N9—C54	118 (3)	C46—C47—N8	125 (3)
C60—N10—C64	117 (3)	C46—C47—H47	117.7
H17C—O17—H17D	109.0	N8—C47—H47	117.7
H18C—O18—H18D	108.0	C53—C48—C49	120 (3)
H19C—O19—H19D	108.8	C53—C48—C35	119 (3)
C2—C1—N1	114 (2)	C49—C48—C35	121 (3)
C2—C1—C6	132 (3)	C50—C49—C48	120 (3)
N1—C1—C6	113 (2)	C50—C49—H49	120.0
C1—C2—C3	121 (2)	C48—C49—H49	120.0
C1—C2—H2	119.5	C51—C50—C49	120 (3)
C3—C2—H2	119.5	C51—C50—H50	120.0
C4—C3—C2	119 (2)	C49—C50—H50	120.0
C4—C3—C16	121 (2)	C50—C51—C52	120 (3)
C2—C3—C16	120 (2)	C50—C51—C54	119 (3)
C5—C4—C3	123 (3)	C52—C51—C54	121 (3)
C5—C4—H4	118.3	C51—C52—C53	120 (3)
C3—C4—H4	118.3	C51—C52—H52	120.0
N1—C5—C4	114 (2)	C53—C52—H52	120.0
N1—C5—C11	113 (2)	C48—C53—C52	120 (3)
C4—C5—C11	133 (3)	C48—C53—H53	120.0
C7—C6—N2	130 (2)	C52—C53—H53	120.0
C7—C6—C1	118 (3)	C51—C54—N9	112 (2)
N2—C6—C1	112 (2)	C51—C54—H54A	109.3
C6—C7—C8	114 (3)	N9—C54—H54A	109.3
C6—C7—H7	122.8	C51—C54—H54B	109.3
C8—C7—H7	122.8	N9—C54—H54B	109.3
C9—C8—C7	119 (3)	H54A—C54—H54B	107.9
C9—C8—H8	120.4	N9—C55—C56	121 (3)
C7—C8—H8	120.4	N9—C55—H55	119.6
C10—C9—C8	121 (3)	C56—C55—H55	119.6
C10—C9—H9	119.3	C55—C56—C57	121 (3)
C8—C9—H9	119.3	C55—C56—H56	119.6
N2—C10—C9	123 (3)	C57—C56—H56	119.6
N2—C10—H10	118.7	C58—C57—C56	117 (3)
C9—C10—H10	118.7	C58—C57—C62	123 (4)
C12—C11—N3	124 (3)	C56—C57—C62	120 (3)
C12—C11—C5	121 (3)	C57—C58—C59	123 (3)
N3—C11—C5	114 (3)	C57—C58—H58	118.3
C13—C12—C11	120 (3)	C59—C58—H58	118.3
C13—C12—H12	120.1	N9—C59—C58	117 (3)
C11—C12—H12	120.1	N9—C59—H59	121.7
C14—C13—C12	119 (3)	C58—C59—H59	121.7
C14—C13—H13	120.4	N10—C60—C61	121 (3)
C12—C13—H13	120.4	N10—C60—H60	119.4
C13—C14—C15	118 (3)	C61—C60—H60	119.4
C13—C14—H14	121.0	C62—C61—C60	118 (3)
C15—C14—H14	121.0	C62—C61—H61	121.0
C14—C15—N3	126 (3)	C60—C61—H61	121.0
C14—C15—H15	116.8	C63—C62—C61	121 (3)
N3—C15—H15	116.8	C63—C62—C57	120 (4)
C17—C16—C21	120 (3)	C61—C62—C57	119 (4)
C17—C16—C3	118 (3)	C64—C63—C62	117 (4)
C21—C16—C3	121 (3)	C64—C63—H63	121.5
C16—C17—C18	120 (3)	C62—C63—H63	121.5
C16—C17—H17	120.0	C63—C64—N10	125 (3)
C18—C17—H17	120.0	C63—C64—H64	117.3
C19—C18—C17	120 (3)	N10—C64—H64	117.3
C19—C18—H18	120.0	N11—C65—C66	171 (6)
C17—C18—H18	120.0	C65—C66—H66A	109.5
C20—C19—C18	120 (3)	C65—C66—H66B	109.5
C20—C19—C22	118 (3)	H66A—C66—H66B	109.5
C18—C19—C22	121 (3)	C65—C66—H66C	109.5
C19—C20—C21	120 (3)	H66A—C66—H66C	109.5
C19—C20—H20	120.0	H66B—C66—H66C	109.5
C21—C20—H20	120.0	N12—C67—C68	165 (9)
C16—C21—C20	120 (3)	C67—C68—H68A	109.5
C16—C21—H21	120.0	C67—C68—H68B	109.5
C20—C21—H21	120.0	H68A—C68—H68B	109.5
C19—C22—N4	109 (2)	C67—C68—H68C	109.5
C19—C22—H22A	109.8	H68A—C68—H68C	109.5
N4—C22—H22A	109.8	H68B—C68—H68C	109.5
C19—C22—H22B	109.8	N13—C69—C70	168 (5)
N4—C22—H22B	109.8	C69—C70—H70A	109.5
H22A—C22—H22B	108.3	C69—C70—H70B	109.5
N4—C23—C24	117 (3)	H70A—C70—H70B	109.5
N4—C23—H23	121.7	C69—C70—H70C	109.5
C24—C23—H23	121.7	H70A—C70—H70C	109.5
C25—C24—C23	125 (3)	H70B—C70—H70C	109.5
C25—C24—H24	117.7	N14—C71—C72	160 (7)
C23—C24—H24	117.7	C71—C72—H72A	109.5
C26—C25—C24	115 (3)	C71—C72—H72B	109.5
C26—C25—C30	125 (3)	H72A—C72—H72B	109.5
C24—C25—C30	121 (3)	C71—C72—H72C	109.5
C25—C26—C27	124 (3)	H72A—C72—H72C	109.5
C25—C26—H26	117.8	H72B—C72—H72C	109.5
C27—C26—H26	117.8		
			
N2—Pt1—N1—C5	−178.3 (16)	C23—C24—C25—C30	177 (3)
N3—Pt1—N1—C5	0.2 (15)	C24—C25—C26—C27	6(5)
Cl5—Pt1—N1—C5	−10 (33)	C30—C25—C26—C27	−176 (3)
N2—Pt1—N1—C1	−4.9 (14)	C23—N4—C27—C26	4(4)
N3—Pt1—N1—C1	173.7 (15)	C22—N4—C27—C26	177 (3)
Cl5—Pt1—N1—C1	164 (31)	C25—C26—C27—N4	−5(5)
N3—Pt1—N2—C10	175 (2)	C32—N5—C28—C29	−11 (4)
N1—Pt1—N2—C10	−180 (2)	N5—C28—C29—C30	11 (5)
Cl5—Pt1—N2—C10	0(2)	C28—C29—C30—C31	−3(5)
N3—Pt1—N2—C6	−4(4)	C28—C29—C30—C25	177 (3)
N1—Pt1—N2—C6	1.2 (15)	C26—C25—C30—C29	−152 (3)
Cl5—Pt1—N2—C6	−178.6 (15)	C24—C25—C30—C29	26 (5)
N2—Pt1—N3—C15	−171 (2)	C26—C25—C30—C31	28 (5)
N1—Pt1—N3—C15	−176 (2)	C24—C25—C30—C31	−154 (3)
Cl5—Pt1—N3—C15	4(2)	C29—C30—C31—C32	−5(5)
N2—Pt1—N3—C11	5(4)	C25—C30—C31—C32	175 (3)
N1—Pt1—N3—C11	0.0 (16)	C28—N5—C32—C31	3(4)
Cl5—Pt1—N3—C11	179.9 (15)	C30—C31—C32—N5	5(5)
N8—Pt2—N6—C33	177 (2)	C37—N6—C33—C34	−9(4)
N7—Pt2—N6—C33	−2(2)	Pt2—N6—C33—C34	175.2 (19)
Cl6—Pt2—N6—C33	−73 (29)	C37—N6—C33—C38	−179 (2)
N8—Pt2—N6—C37	1.4 (17)	Pt2—N6—C33—C38	6(3)
N7—Pt2—N6—C37	−177.7 (18)	N6—C33—C34—C35	9(4)
Cl6—Pt2—N6—C37	111 (29)	C38—C33—C34—C35	176 (3)
N6—Pt2—N7—C42	−175 (2)	C33—C34—C35—C36	−7(4)
N8—Pt2—N7—C42	−177 (2)	C33—C34—C35—C48	180 (2)
Cl6—Pt2—N7—C42	4(2)	C34—C35—C36—C37	6(4)
N6—Pt2—N7—C38	−2.7 (18)	C48—C35—C36—C37	180 (2)
N8—Pt2—N7—C38	−6(4)	C35—C36—C37—N6	−7(4)
Cl6—Pt2—N7—C38	175.5 (17)	C35—C36—C37—C43	180 (2)
N6—Pt2—N8—C43	−0.9 (16)	C33—N6—C37—C36	8(3)
N7—Pt2—N8—C43	2(3)	Pt2—N6—C37—C36	−176.1 (17)
Cl6—Pt2—N8—C43	−179.2 (15)	C33—N6—C37—C43	−177 (2)
N6—Pt2—N8—C47	−180 (2)	Pt2—N6—C37—C43	−2(3)
N7—Pt2—N8—C47	−177 (2)	C42—N7—C38—C39	−4(4)
Cl6—Pt2—N8—C47	2(2)	Pt2—N7—C38—C39	−177 (2)
C5—N1—C1—C2	−8(3)	C42—N7—C38—C33	179 (2)
Pt1—N1—C1—C2	179.1 (15)	Pt2—N7—C38—C33	6(3)
C5—N1—C1—C6	179.9 (19)	N6—C33—C38—C39	176 (3)
Pt1—N1—C1—C6	7(2)	C34—C33—C38—C39	7(5)
N1—C1—C2—C3	5(3)	N6—C33—C38—N7	−7(3)
C6—C1—C2—C3	175 (2)	C34—C33—C38—N7	−176 (3)
C1—C2—C3—C4	−4(3)	N7—C38—C39—C40	2(4)
C1—C2—C3—C16	177 (2)	C33—C38—C39—C40	179 (2)
C2—C3—C4—C5	4(4)	C38—C39—C40—C41	3(4)
C16—C3—C4—C5	−176 (2)	C39—C40—C41—C42	−7(4)
C1—N1—C5—C4	9(3)	C38—N7—C42—C41	0(4)
Pt1—N1—C5—C4	−179.1 (15)	Pt2—N7—C42—C41	172 (2)
C1—N1—C5—C11	−173 (2)	C40—C41—C42—N7	5(4)
Pt1—N1—C5—C11	0(2)	C47—N8—C43—C44	−1(3)
C3—C4—C5—N1	−6(3)	Pt2—N8—C43—C44	−180 (2)
C3—C4—C5—C11	176 (2)	C47—N8—C43—C37	179.3 (19)
C10—N2—C6—C7	7(3)	Pt2—N8—C43—C37	0(3)
Pt1—N2—C6—C7	−174.0 (19)	C36—C37—C43—C44	−5(4)
C10—N2—C6—C1	−176.8 (18)	N6—C37—C43—C44	−179 (2)
Pt1—N2—C6—C1	2(2)	C36—C37—C43—N8	175 (2)
C2—C1—C6—C7	1(4)	N6—C37—C43—N8	1(3)
N1—C1—C6—C7	170.5 (19)	N8—C43—C44—C45	5(4)
C2—C1—C6—N2	−176 (2)	C37—C43—C44—C45	−175 (2)
N1—C1—C6—N2	−6(3)	C43—C44—C45—C46	−10 (4)
N2—C6—C7—C8	−6(4)	C44—C45—C46—C47	11 (4)
C1—C6—C7—C8	178 (2)	C45—C46—C47—N8	−7(4)
C6—C7—C8—C9	2(3)	C43—N8—C47—C46	2(3)
C7—C8—C9—C10	0(4)	Pt2—N8—C47—C46	−179.2 (18)
C6—N2—C10—C9	−4(3)	C34—C35—C48—C53	−171 (3)
Pt1—N2—C10—C9	176.8 (17)	C36—C35—C48—C53	15 (4)
C8—C9—C10—N2	1(4)	C34—C35—C48—C49	13 (4)
C15—N3—C11—C12	−1(3)	C36—C35—C48—C49	−160 (3)
Pt1—N3—C11—C12	−177.7 (19)	C53—C48—C49—C50	0(4)
C15—N3—C11—C5	176.3 (19)	C35—C48—C49—C50	175 (2)
Pt1—N3—C11—C5	0(3)	C48—C49—C50—C51	0(4)
N1—C5—C11—C12	178 (2)	C49—C50—C51—C52	0(4)
C4—C5—C11—C12	−4(4)	C49—C50—C51—C54	−172 (3)
N1—C5—C11—N3	0(3)	C50—C51—C52—C53	0(5)
C4—C5—C11—N3	179 (2)	C54—C51—C52—C53	172 (3)
N3—C11—C12—C13	1(4)	C49—C48—C53—C52	0(4)
C5—C11—C12—C13	−176 (2)	C35—C48—C53—C52	−176 (2)
C11—C12—C13—C14	−3(4)	C51—C52—C53—C48	0(4)
C12—C13—C14—C15	4(4)	C50—C51—C54—N9	−108 (3)
C13—C14—C15—N3	−4(4)	C52—C51—C54—N9	81 (4)
C11—N3—C15—C14	3(3)	C55—N9—C54—C51	96 (4)
Pt1—N3—C15—C14	178.3 (18)	C59—N9—C54—C51	−89 (3)
C4—C3—C16—C17	168 (2)	C59—N9—C55—C56	4(5)
C2—C3—C16—C17	−12 (4)	C54—N9—C55—C56	179 (3)
C4—C3—C16—C21	−7(4)	N9—C55—C56—C57	1(5)
C2—C3—C16—C21	173 (2)	C55—C56—C57—C58	−5(5)
C21—C16—C17—C18	0(4)	C55—C56—C57—C62	−178 (3)
C3—C16—C17—C18	−175 (2)	C56—C57—C58—C59	6(5)
C16—C17—C18—C19	0(4)	C62—C57—C58—C59	178 (3)
C17—C18—C19—C20	0(5)	C55—N9—C59—C58	−4(4)
C17—C18—C19—C22	173 (3)	C54—N9—C59—C58	−179 (2)
C18—C19—C20—C21	0(5)	C57—C58—C59—N9	−1(5)
C22—C19—C20—C21	−174 (3)	C64—N10—C60—C61	−3(5)
C17—C16—C21—C20	0(4)	N10—C60—C61—C62	0(5)
C3—C16—C21—C20	175 (2)	C60—C61—C62—C63	0(5)
C19—C20—C21—C16	0(5)	C60—C61—C62—C57	180 (3)
C20—C19—C22—N4	−96 (3)	C58—C57—C62—C63	160 (3)
C18—C19—C22—N4	90 (3)	C56—C57—C62—C63	−28 (5)
C27—N4—C22—C19	80 (3)	C58—C57—C62—C61	−20 (5)
C23—N4—C22—C19	−108 (3)	C56—C57—C62—C61	152 (3)
C27—N4—C23—C24	−4(4)	C61—C62—C63—C64	3(5)
C22—N4—C23—C24	−176 (3)	C57—C62—C63—C64	−176 (3)
N4—C23—C24—C25	4(5)	C62—C63—C64—N10	−7(5)
C23—C24—C25—C26	−5(5)	C60—N10—C64—C63	7(5)
